# Correction: Community-orientated primary health care: Exploring the interface between community health worker programmes, the health system and communities in South Africa

**DOI:** 10.1371/journal.pgph.0002365

**Published:** 2023-09-01

**Authors:** The PLOS Global Public Health Staff

There are errors in the Funding statement. The publisher apologizes for the errors. The correct Funding statement is as follows: The study was supported by Medical Research Council UK, Department of International Development (DFID), Economic and Social Research Council (ESRC) and Wellcome Trust under the Joint Health Systems Research Initiative (JHSRI) (Grant number: MR/NO15908) awarded to JG. It was also supported by the South African National Research Foundation (NRF) through the South African Research Chair (SARChi) in Health Systems and Policy awarded to the Centre for Health Policy, University of the Witwatersrand, and held by FG. All three authors received salary supplementation as part of the research funding from JHSRI. The specific roles of these authors are articulated in the ‘author contributions’ section. The funders had no role in study design, data collection and analysis, decision to publish, or preparation of the manuscript.

## References

[1] Malatji H, Griffiths F, Goudge J (2023) Community-orientated primary health care: Exploring the interface between community health worker programmes, the health system and communities in South Africa. PLOS Glob Public Health 3(2): e0000881. 10.1371/journal.pgph.0000881 36962793 PMC10021906

