# Supplementary material for: Community-orientated primary health care: Exploring the interface between community health worker programmes, the health system and communities in South Africa
Source: PLOS Glob Public Health. 2023 Feb 14;3(2):e0000881. doi: 10.1371/journal.pgph.0000881 (PMC10021906; doi:10.1371/journal.pgph.0000881)
Supplement: S1 Checklist — (DOCX) [file pgph.0000881.s001.docx]

**COREQ (Consolidated criteria for Reporting Qualitative research) Checklist**

| **topic** | **Item No** | **Guide question/description** | **Reported on page No** |
| --- | --- | --- | --- |
| **Domain 1: Research team and reflexivity** | | | |
| *Personal characteristics* | | | |
| Interviewer/facilitator | 1 | Which author/s conducted the interview or focus group? | Methods, page 9 and 10, paragraph 1 |
| Credentials | 2 | What were the researcher’s credentials? E.g. PhD, MD | Methods, page 10, paragraph 1 |
| Occupation | 3 | What was their occupation at the time of the study? | Methods, page 9, paragraph 1 |
| Gender | 4 | Was the researcher male or female? | Methods, page 9, paragraph 1 |
| Experience and training | 5 | What experience or training did the researcher have? | Methods, page 9, paragraph 1 |
| *Relationship with participants* | | | |
| Relationship established | 6 | Was a relationship established prior to study commencement? | Methods, page 5-6, paragraph 4  Page 6, paragraph 2 |
| Participant knowledge of the interviewer | 7 | What did the participants know about the researcher? e.g. personal goals, reasons for doing the research | Methods, page 9, paragraph 1 |
| Interviewer characteristics | 8 | What characteristics were reported about the interviewer/facilitator? e.g. Bias, assumptions, reasons and interests in the research topic | Methods, page 9, paragraph 1 |
| **Domain 2: Study design** | | | |
| *Theoretical framework* | | | |
| Methodological orientation and Theory | 9 | What methodological orientation was stated to underpin the study? e.g. grounded theory, discourse analysis, ethnography, phenomenology, content analysis | Methods, page 6, paragraph 2 |
| *Participant selection* | | | |
| sampling | 10 | How were participants selected? e.g. purposive, convenience, consecutive, snowball | Methods, page 5, paragraph 4 |
| Method of approach | 11 | How were participants approached? e.g. face-to-face, telephone, mail, email | Methods, page, paragraph 1 |
| Sample size | 12 | How many participants were in the study? | Methods, table 10, page 10 |
| Non-participation | 13 | How many people refused to participate or dropped out? Reasons? | Methods, page 9, paragraph 3 |
| *Setting* | | | |
| Setting of data collection | 14 | Where was the data collected? e.g. home, clinic, workplace? | Methods, page 7, paragraph 1 |
| Presence of non-participant | 15 | Was anyone else present besides the participants and researchers? | Methods, page 9, paragraph 3 |
| Description of sample | 16 | What are the important characteristics of the sample? e.g. demographic data, date? | Findings, page 11, paragraph 2 |
| *Data collection* | | | |
| Interview guide | 17 | Were questions, prompts, guides provided by the authors? Was it pilot tested? | Method, page 9, paragraph 2-4 |
| Repeat interviews | 18 | Were repeats interviews carried out? If yes, how many? | Methods, page 9, table 1 |
| Audio/visual recording | 19 | Did the research use audio or visual recording to collect the data? | Method, page 9, paragraph 1 |
| field notes | 20 | Were field notes made during and/or after the interview or focus group? | Method, page 9, paragraph 3 |
| Duration | 21 | What was the duration of the interviews or focus group? | Method, page 9, paragraph 2 and 4 |
| Data saturation | 22 | Was data saturation discussed? | Methods, paragraph 2, page 10 |
| Transcripts returned | 23 | Were transcripts returned to participants for comment and/or correction | Methods, paragraph 2, Page 10? |
| **Domain 3: analysis and findings** | | | |
| *Data analysis* | | | |
| Number of data coders | 24 | How many data coders coded the data? | Methods, Page 10, paragraph 1 |
| Description of coding tree | 25 | Did authors provide a description of the coding tree? | Method,  page 10, paragraph 1 |
| Derivation of themes | 26 | Were themes identified in advance or derived from the data? | Method, page 10, paragraph 1 |
| software | 27 | What software, if applicable, was used to manage the data? | Method, page 10, paragraph 1 |
| Participant checking | 28 | Did participants provide feedback on the findings? | Method, page 5, paragraph 2 |
| *Reporting* | | | |
| Quotations presented |  | Were participant quotations presented to illustrate the themes/findings? Was each quotation identified? e.g. participant number | Findings, page 11-21 |
| Data and findings consistent | 30 | Was there consistency between the data presented and the findings? | Findings, page 11-21 |
| Clarity of major themes | 31 | Were major themes clearly presented in the findings? | Findings, page 13-21 |
| Clarity of minor themes | 32 | Is there a description of diverse cases or discussion of minor themes? | Findings, page 11-13 |
