# Supplementary material for: Community-orientated primary health care: Exploring the interface between community health worker programmes, the health system and communities in South Africa
Source: PLOS Glob Public Health. 2023 Feb 14;3(2):e0000881. doi: 10.1371/journal.pgph.0000881 (PMC10021906; doi:10.1371/journal.pgph.0000881)
Supplement: Attachment — Submitted filename: Response to reviewers comments.docx [file pgph.0000881.s002.docx]

**Dear Shailendra Prasad**

PLOS Global Public Health

**Re: authors responses to editor and reviewers’ comments**

Thank you for the feedback on 22 August 2022, and for the opportunity to resubmit the revised manuscript to the journal. We went through feedback from the six reviewers and found the feedback helpful. Please see the reviewers’ comments and our responses in the below table

| **Editor** | |
| --- | --- |
| **Comments** | **Author responses** |
| 1. Please amend your detailed Financial Disclosure statement. This is published with the article. It must therefore be completed in full sentences and contain the exact wording you wish to be published. | The financial disclosure section has been amended to read as follows:  “The study was funded by Medical Research Council UK, DFID, ESRC and Wellcome Trust under the Joint Health Systems Research Initiative (JHSRI). The funders were not involved in the study design, data collection, analysis and preparation of this manuscript. All three authors received salary supplementation as part of the research funding from JHSRI.” |
| 2. State what role the funders took in the study. If the funders had no role in your study, please state: “The funders had no role in study design, data collection and analysis, decision to publish, or preparation of the manuscript.” | Please see text above |
| 3. If any authors received a salary from any of your funders, please state which authors and which funders. | Please see text above |
| 4. In the online submission form you indicate that your data is not available for proprietary reasons and have provided a contact point for accessing this data. Please note that your current contact point is a co-author on this manuscript. According to our Data Policy, the contact point must not be an author on the manuscript and must be a third party. Please revise your data statement to a non-author institutional point of contact, such as a data access or ethics committee, and send this to us via return email. Please also include contact information for the third-party organization, and please include the full citation of where the data can be found. | As an institution in a LMIC, the University of the Witwatersrand’s central research office, or the Ethics committee does not have sufficient funds to run a central data repository. Moreover, due to the importance of understanding the context in which qualitative data was collected, we think it is important for the researchers to be the point for contact for whoever wishes to analyse the data; that way we can ensure the relevance of the research question, and that the data isn’t mis-interpreted. We would be interested to know how your other authors of qualitative papers based in LMICs have resolved this issue. |
| 5. We have amended your Competing Interest statement to comply with journal style. We kindly ask that you double check the statement and let us know if anything is incorrect. | Statement is correct:  “The authors have declared that no competing interests exist” |
| **Reviewer 1** | |
| **Comments** | **Authors responses** |
| 6. There are several confusions that the authors must clarify. 1) When the authors write , “However, many residents were more concerned about access to housing that health services delivery issues” I am not sure whether the authors are referrin” to the patients of the CHW. In the sentence quoted above do they mean, access to housing than health services. If yes, then what is the implication of this? That CHW were not perceived as important on the field by the residents? IF so, then this must be emphasized as the gap between consensus in public health about the importance of CHWs and service recipients who see other public goods as important such as housing. | Yes, the statement that ‘many residents (ie not just patients) were more concerned with housing than health care service delivery’ is correct. This means at public meetings residents weren’t interested in discussing health care, because the lack of housing had such dire consequences for the community; in contrast the delivery of health care worked relatively well.  The text has been clarified and sentence added to further explain residents’ relationship with the CHW programme, it currently read as follows:  “One of the key principles of COPC is for health programme developers to engage communities and ensure the services to be provided are relevant to the needs of the community. It is important that there are open channels of communication such that if there is a misunderstanding, issues can be clarified.”  We have added some text in the paper to explain the importance of engagement with the community as a collective, and the relative of importance of housing and health care to the community. In the discussion, we pick on this in more detail. |
| 7. When the authors write, "Governments need to provide sufficient funds for training, supervision, supplies and remuneration to help overcome these barriers" they are not paying heed to the history of CHWs. It was precisely because of various lacks and scarcities were CHWs seen as important. Are there are cost analysis studies being done? For example, look at Sarang Deo's work. | We agree that there is a shortage of resources in the health system and so generate the need for CHW services. However, the shortage of resources is also the case within CHW programmes.  We did not do a cost analysis within this study. |
| 8. Finally, please look at the work of Svea Closser, Alex Nading, on the kind of work CHWs do and the kind of figure they are in the structures of national public health. | We went through the work of the identified scholars. We discuss the pertinent role of CHWs in healthcare, especially how CHWs serve as brokers between communities and healthcare system. Furthermore, Nading et al also describes how CHWs are always made to take responsibility for the inefficiencies of the healthcare system. These issues are also observable within the South African healthcare system, and we have described them in other articles:   - Tseng Y, Griffiths F, de Kadt J, et alIntegrating community health workers into the formal health system to improve performance: a qualitative study on the role of on-site supervision in the South African programmeBMJ Open 2019;9:e022186. doi: 10.1136/bmjopen-2018-022186 - Anstey Watkins J, Griffiths F, Goudge JCommunity health workers’ efforts to build health system trust in marginalised communities: a qualitative study from South AfricaBMJ Open 2021;11:e044065. doi: 10.1136/bmjopen-2020-044065 |
| **Reviewer 2** | |
| **Comments** | **Authors responses** |
| 9. Abstract – the abstract seems more general. Authors can write more specifically about south Africa from the beginning, including the multidisciplinary approach and the socioeconomic barriers are? | Thank you for the feedback.  When a paper focuses on a single country’s experience, our strategy is to first identify the international issue at stake, and then explain the specifics of the country on which we will focus. In this way, we can illustrating how that country’s experience will provide useful lessons and understanding that is relevant to the international problem that has been outlined. We are of the view that it is important to link an individual country’s experience to broader debates and structuring the abstract in this way allows us to do this. |
| 10. The authors seem more focussed on COPC as the paper's main argument, therefore mentioned in the title. The current title looks more methodological paper. That is clearly evident in the framing of the introduction. Authors can reframe the introduction section with the beginning of the CHWs programs (specific) and the South African health system context, where some research gaps can be justified. Authors can address those research gaps using the COPC approach as a method, including other methods used in the research. | Thank you for the feedback.  The researchers revisited the title and found no mention of methodological issue. Community-orientated primary healthcare is an approach or framework that provides guidance as to how to implement of primary health care programmes; it is not a research methodology. Given this misunderstanding, we have chosen not to reframe the introduction. |
| 11. As the authors mentioned as a case study as a methodological design, some explanation of how it is case study design is appropriate for this could be important to justify the methodological justification. | The study design text has been amended:  “Drawing on data collected as part of a larger CHW study [15] and doctoral research, we used a descriptive case study approach involving multiple qualitative methods to examine seven CHW teams. The use of case study design is suitable when researchers want to gain concrete, contextual and in-depth knowledge of a phenomenon. The design is used to explore key features of a particular phenomenon. In the current study, the design enabled us to examine the practice of COPC in the context of the nation-wide CHW programme, CHWs relationship with the community and integration with the health system [16, 17]” |
| 12. Regarding the data collection and numbers of participants, the authors might have selected them using information saturation, which can be explained to demonstrate why the authors selected specific numbers of participants. | Thank you for the comment. In our understanding, saturation is not used to select participants, rather to determine when, if using snow-ball sampling strategy for example, to stop data collection.  For the CHW teams, the researchers interviewed all participants who formed part of the team. Where the CHWs numbers exceeded the number suitable for a focus group discussion, the researchers held two focus groups to accommodate the excess.  Community representatives and key informants were selected based on availability and programme knowledge.  We have edited the text to provide more of these details in the text. |
| 13. Data analysis is unclear whether it is thematic analysis or framework analysis. The authors indeed used a deductive approach to fit contents in the COPC framework. Thus, more clarity is needed here. | The data analysis section has been edited to make this clear, and it now reads as follows:  “We used inductive approach to identify themes emerging from the data, as well as framework analysis. The first author used a MS word sheet to extract data from the interview transcripts and observation notes from the seven study sites. The information was summarised while retaining important quotations. The author regularly presented the extracted data to the co-authors; this was done to ensure completeness of the data and that all relevant information was being extracted. The resulting themes were group into broader COPC themes i.e. the nature of community-based health care services, the challenges CHWs encounter in providing services and evidence of COPC principles in the different sites. The themes were compared for similarities and differences in the different sites. The themes were synthesised to understand the history of the programmes, type of services provided by the CHWs, funding and socioeconomic issues of the communities. Moreover, evidence of COPC principles was also extrapolated from the different programmes”. |
| 14. The first part (site-specific explanation of findings ) of the result section is redundant with the latter part of the findings. The first part can be summarised in the table with a key description. | We are not convinced that the first part of the results section is redundant as it describes the differences and similarities of the programmes and the communities that they serve. However, we do agree that a table that summaries the key points for ease of reference, would be helpful. (Also see response to point 26 below) |
| 15. It is unclear what themes emerged in the data analysis in each COPC component and what supporting evidence for these themes. The authors provided a long text of paragraphs, including verbatim and descriptions. Authors can write themes under each COPC component. That might be more structured and explanatory. More analysis of the data is needed. | We understand this point to be referring to the fact that under the COPC component of service integration, we identified some subthemes (distance / location, supervision, relationships with health care workers), but not in others. We understand the reviewer is requesting that we identify the sub-themes of each COPC principle. We have added subthemes for some of the principles with longer text, however for the shorter sections, we did not add the sub-themes as we run the risk of cutting up the text into sections that are too small. We have reviewed these sections for clarity. |
| 16. There is good content in the discussion section; however, the writing structure made it difficult to follow. In the first para of the discussion section, the authors can summarise key overarching findings that can be discussed in subsequent paragraphs using the similar framework used in the finding section. So, from the contents and structure point of view, this section required more revision to draw the key implications for the policy and programs regarding CHWs programs in SA. | Thank you for the feedback on the discussion.  The first paragraph as suggested by the reviewer, summarises the findings the evidence of COPC principles being used in the CHW programmes, as well as how the socio-economic conditions that hamper the delivery of health care. We have strengthened this paragraph.  Rather than repeating the structure of the results section in the discussion, we have picked four issues that have implications for policy and practice, and further research, to discuss in greater depth. We have ensured that each paragraph starts with a topic sentence to help indicate to the reader the focus of each paragraph, and so how the section is structured. We outline the paragraphs here.  The second paragraph describes the CHW poor skills as a limitation of the programmes, and how in-service training can be used to improve the knowledge and skills of the CHWs to provide comprehensive care.  Paragraph three describes the over reliance on external funds to implement CHW programmes as a limitation. Since when the funding ends, the programmes stall.  Paragraph four, we argue that while community participation is important in the design, development and implementation of CHW programmes, it was lacking in our communities. This is because the lack of housing was of prime importance for community members, and health care was of secondary importance.  In paragraph five, we discuss community health system as another approach to deliver community-based health care.  Paragraph six highlights the study limitations and strengths. |
| 17. Authors can include limitations of the study and section programs for policy and programs too. | The discussion section includes a section on the study limitations, kindly see paragraph 6  There is reference to policy and programme design and implementation. Kindly see paragraph 3, 4 and 5. |
| 18. The current conclusion section is general. Authors can include key points. The first line of the conclusion is your method/approach, not conclusions. The conclusion should be made based on the findings discussed in the paper. More specific conclusions are needed. | Thank you for the feedback, as mentioned under point 11 above, COPC is not a methodological approach, it is rather a framework used to guide the implementation of primary health care. We have made additions to the conclusion to accommodate other topics in the discussion:  “The implementation of community-orientated primary health care services is crucial in a resource constrained setting like South Africa. However, the implementation of these programmes often gets derailed by socio-economic issues dominant in the communities. Poor community participation in the design and implementation of the programmes, limited health system support for outreach teams and unsustained funds make it difficult for the teams to be effective in the delivery of care. In order to strengthen these programmes, CHWs need to be integrated into healthcare systems so to access supportive supervision, have access to resources, and governments make available funding to implementing teams.” |
| **Reviewer 3** | |
| **Comments** | **Authors responses** |
| 19. There are some issues regarding the clarity of objective and research methodology that could help improve the paper   In the methods, the authors state that they used “a descriptive case study approach”. Could the authors please give a bit more details so that it sets the reader up a bit better?. Perhaps stating clearly that multiple qualitative methods will be used?. | We have revised the text on our research methodology which now reads as follows. (This is also described in response to point 11 above.)  “Drawing on data collected as part of a larger CHW study [15] and doctoral research, we used a descriptive case study approach involving multiple qualitative methods to examine seven CHW teams. The use of case study design is suitable when researchers want to gain concrete, contextual and in-depth knowledge of a phenomenon. The design is further used to explore key features of a particular phenomenon. In the current study, the design enabled us to examine the practice of COPC in the context of the nation-wide CHW programme, CHWs relationship with the community and integration with the health system [16, 17]” |
| 20. The objective to examine “the extent” of community orientation of program is also a bit vague. How can the “extent” of quantified or summarized? | We agree this is an important question. However, no previous studies as far as we are aware have developed an approach that would allow quantifying the COPC principles. We didn’t attempt to do this either. We suspect that it would complex, and presumably any numeric criteria would vary across settings. We have mentioned this issue as one of the limitations of the study |
| 21. The clarity of quantitative analysis could improve. As written, the authors have used the COPC principles as their main headings. Some headings have themes, but some do not. For example, for the heading “service integration” there are 4 themes (distance/location, supervision, relationships, and referral systems) but for example, for “comprehensive and multi-disciplinary care” and “community participation”, there are no themes, but based on the descriptive given, some themes may have emerged. | We presume you mean ‘clarity of qualitative analysis’ rather than quantitative?  As described above in response to point 15, we have now added themes to comprehensive care and multidisciplinary approach to care, as these were the longest sections. For the shorter sections, we did not add the themes as we run the risk of cutting up the text into sections that are too small. We have reviewed these sections for clarity. |
| 22. Along with my previous comment, please visit the COREQ checklist or SQRQ checklist so that all qualitative components are adequately described and summarized.  I could not access the observation template, but as there were close to 100 observations, perhaps some of the observations made can be summarized quantitatively. | We have made edits to the manuscripts to ensure compliance with the COREQ checklist. We added text to population and sample, and data collection section of the paper. Kindly find attached completed COREQ checklist  This data form part of a larger project on CHWs in South Africa. Our other paper has quantified the observations.   - Goudge J, de Kadt J, Babalola O, et al Household coverage, quality and costs of care provided by community health worker teams and the determining factors: findings from a mixed methods study in South AfricaBMJ Open 2020;10:e035578. doi: 10.1136/bmjopen-2019-035578 |
| **Reviewer 4** | |
| **Comments** | **Authors responses** |
| 23. Study sites Although there is clarity regarding the diversity of the 4 districts selected, it is unclear as to why these 4 districts were selected. It appears like there are 52 districts in South Africa, and these 4 districts (Sedibeng, Johannesburg, Tshwane and Ehlanzeni) seem to be geographically close to each other. This might have implications on extrapolating the experience to the nation-wide program. | We have reviewed the text on study sites and made some edits to the study sites. Kindly see the edited text below:  “The case studies / CHW teams were purposively selected based on their location, history and model of adopting the CHW programmes. The unique teams provided rich comparative case studies. The distance between Gauteng and Mpumalanga facilities was approximately 500kms.”  Mpumalanga based programmes were included as they have a reputation of being well run; we also anticipated that the differences between urban or semi-urban, and rural sites would be important, particularly in trying to deliver a community orientated programme. |
| 24. Further, it isn’t clear whether the government program has three different models. Table 2 talks about “Gvt programme; no additional funding”, “Gvt programme with additional external funding”, and “Gvt funding but sub-contracted to local NGOs”. Later in the discussion section, 464-468 lines: “In our study, the COPC initiatives studied relied heavily on external resources to recruit, train and retain the CHWs, and unfortunately, when the funds ran out the initiatives stalled. Many of those writing about community-based programme argue sustainable initiatives require financial commitment from government”. Is the analysis about the nation-wide program or on the different CHW programs that has been running in the 4 districts? | Thank you for this feedback.  The South African government introduced the nation-wide CHW programme in 2011. Prior to the nation-wide programme, some of the study sites had implemented CHW programmes with a COPC focus, some employed a COPC focus as they implemented the nation-wide programme. We have reviewed the manuscript to clarify the focus of the study in relation to the programmes. The study purpose in the background has been edited to read as follows “The aim of the paper was to examine whether the programmes as they existed at the time of the fieldwork (2018-19 ie 7-8 years post the nation-wide rollout) were implemented in accordance with the principles of COPC”. |
| 25. This question on what is being evaluated made me, as a reader, confused when reading the “Findings” section. For example in 204-206 lines, the authors say “However, with the introduction of the nation-wide CHW programme in 2011, the programme was experiencing challenges, as funds and other institutional resources were diverted to the nation-wide CHW programme”. Which program was observed by the team in Sedibeng? The nation-wide CHW program or the one that was introduced in 2010? Which one is the analysis about? Did the programs get merged in 2011? As this reviewer is not well-read on CHW programs in South Africa, it would help to have a clarifying explanation. | As described in the response above, the South African government introduced the nation-wide programme in 2011. Some of the districts were implementing COPC focused CHW programmes, prior to the introduction of the national programme, one added COPC activities to the nation-wide programme as the latter was rolled out. Our evaluation question was to what extent were the programmes, as they existed given their various histories, being implemented using the COPC principles.  To make this clear in the paper, we have edited the purpose statement of introduction to clearly reflect this. See our response to the comment 24. |
| 26. Similar confusion occurs to this reviewer on reading the history in Johannesburg.  It could be that the story of origin of these programs are intricately tied to how they operated and integrated with COPC principles. In that case, it might be useful to divide the findings section differently. In the present manuscript the findings are divided based on COPC principle into “Assessment of local needs and assets and use of evidence”, “Service Integration”, etc. While this is valuable, this division merges the stories of the four study sites together. I was forced to frequently jump back and forth between the “THE CHW PROGRAMMES AND THE COMMUNITIES THEY SERVE” section and the “COMMUNITY ORIENTATION OF THE CHW PROGRAMMES” section to understand which site has which characteristics. Again, this could be due to my ignorance about South Africa. But considering this is an international journal, I propose an alternative way of grouping the findings.   The findings could be grouped according to the sites. It would be very interesting to read “The experience from Sedibeng”, “The experience from Johannesburg”, “The experience from Tshwane”, and “The experience from Ehlanzeni” as four stories. This would help the reader understand the unique characteristics and history of each site, the rural/urban nature of the site, the funding mechanisms, the way the program was rolled out there, and the way it is practised there. This seems like a less confusing way to organize the findings. The authors may consider ignoring this idea if it leads to difficulties in organizing the findings within the story of each site. | Thanks for this comment. We agree it is important to make the paper easier to understand for non-South African readers. We hope that the addition of the table summarising the communities in the first results section will help, by providing an easier way for the reader to remind him/herself about the different communities. (See response to comment 14 above). If we were to split up the second section of the findings and present the evidence of COPC focus for each of the teams separately, we would still be left with the problem of how to compare, for example, the ‘use of evidence’ across the different teams. We understand the reviewer to be referring to this problem at the end of his/her comment.  . |
| 27. The first paragraph of the discussion section (436-446) could be dissolved into the preceding section. If the authors so wish, it might be possible to merge the whole discussion section into the stories above by highlighting and comparing the relevant literature in the corresponding paragraphs within the story of each site. But if that’s too disruptive, the discussion reads fine as it is. | The accepted format of most journals is to have a separate discussion section. We think this is important to allow reflection on the findings, their situation in other literature, and drawing out implications for policy and practice. |
| 28. **A few sentences/statements/observations struck me as lacking nuance:**  44-46: Due to insufficient number of health workers, community health workers (CHW) are being deployed to provide health care services to under-served communities[3]  The reference doesn’t substantiate the claim that CHWs are being deployed due to insufficient number of health workers. CHWs are health workers. They have their own independent role that cannot be fulfilled by medical practitioners (nurses or doctors). The sentence as it stands makes it appear like CHWs are filling the vacuum of other health workers and provide some sort of weakened, diluted form of care than what would have been possible if there were a sufficient number of (other) health workers. | We agree with the reviewer that the sentence needs further editing and elaboration. It has been edited in the manuscript to read as follows:  “In response to the call for universal health coverage (UHC) and limited number of health workers, many low and middle-income countries (LMICs) are strengthening the delivery of primary health care (PHC) services using community health workers (CHWs) [1, 2]. CHW programmes gained prominence post the Alma Ata Conference of 1978, which prioritised the strengthening of primary healthcare to provide care to marginalised communities. Many large-scale CHW programmes often led by non-government organizations (NGOs) were introduced to provide the care in LMICs [1]. However due to insufficient investment and fragmentation, the performance of these programmes declined during the 1980s. However,, there is now considerable evidence of the expansion and benefits of CHW programmes, particularly for the delivery of maternal and child health services [3, 4]. Reviews show CHW programmes need to be integrated with the community to be attuned to their needs, as well as integrated into the health services to be well-resourced, well supervised, and to communicate their community’s needs to practitioners and decision makers [5, 6]. There has been a growing interest in community-orientated primary care (COPC). COPC is an approach to health service development and delivery that integrates public health and primary care to deliver targeted services to a defined community [6]. This approach recognises the roles of lay health workers, civil society, non-governmental organisations and government sectors in the planning, designing and delivery of primary health care.” |
| 29. 437-439: There was some evidence of resistance by community members to participate in the design and implementation of the health programmes, as the residents were more interested in housing than healthcare.  Housing *is* healthcare. This judgement on the residents is perhaps antithetical to COPC. | The reviewer raised a very important point.  We are not judging the residents; this data emanates from our observation of the residents during community meetings called by local political leaders. In these meetings, the residents did not want to talk about the delivery of healthcare but rather access to housing and were very vocal about their housing needs. This led us to conclude the residents were not interested in healthcare but other needs such as housing. |
| 30. The abstract includes a typo: “However, many residents were more concerned about access to housing that health services delivery issue” - “that” should be “than” | Sentence corrected |
| 31. 440: “particularly were the CHWs” should be “particularly where the CHWs” | “Were” has been replaced with “where” |
| **Reviewer 5** | |
| **Comments** | **Authors responses** |
| 32. Abstract Discuss the timespan of the CHW program under study (following UHC efforts in 2011); otherwise, the reader does not know if this is a decades-long program or a new initiative. | We have added CHW programmes timeframe. The aim of the article now read as follows:  “In this article, we explore to what extent the South African CHW programmes introduced between 2009 and 2011 are attuned to community needs, integrated into the healthcare system and community structures, and also implemented in accordance with community-orientated primary health care principles” |
| 33. Background/Introduction Please provide a brief (1 well-cited paragraph) on the history of CHWs in global health - this has been a key strategy since the 1950s, gained prominence following Alma-Ata, and has had renewed energy through UHC. This will help connect your work and the experience in South Africa to global trends. | We have strengthened the introduction to include one paragraph on the history the CHW programmes pre and post the Alma Ata conference of 1978. This paragraph is included in the response to comment 28 above. |
| 34. Further discussion of and citation of the huge CHW literature is vital for positioning your work.  You must define CHWs for your study. There is a very wide range of what is defined as a CHW globally, and we need a clear picture from the outset exactly what kind of group you are describing. In the background, you can describe the CHW program "on paper" - what is intended? How are CHWs selected/recruited? (We only learn in the Discussion (line 474) that communities have no involvement in selection.) Are CHWs paid? What is the scope of the role - what kinds of tasks? Is it full-time? Ideally, how are CHWs trained and supervised? We need to know all of this to then be able to understand program goals and compare to the reality of what is actually happening as presented in the results. | As per point 34, we have strengthened the introduction with literature on CHW programmes.  CHWs in our study are defined, information about their contractual arrangement and remuneration has been added to the text:  The WBOTs comprise at least 6 CHWs, linked to a local PHC facility, serving a defined geographic area, providing a combination of promotive and preventative healthcare services to households, and making referrals to social services workers [13]. The WBOT team is meant to be led by an outreach team leader who is a nurse and supported by a health promoter and an environmental officer. The role of the outreach team leader is to provide field supervision to the CHWs during household visits, ensure the CHWs have the resources that they require (e.g. stationary) to fulfil their duties, and help establish team relationship with community structures. The CHWs receives training on identification of the need for antenatal and post-natal care, monitoring immunization and adherence to long-term medication, screening for malnutrition, TB, gender-based violence, making referrals to health and social services, and following up on patients who need to visit the health facility. The training is delivered in two phases, the first with a written examination (level 1), the second with a practical assessment in the field (level 2). At the time of the study, the CHW worked 6 hours per day, on a 12 months renewable contract earning a monthly stipend of R3 500 (201 USD). |
| **Methods** 35. Note in Table 3 the number of participants in each focus group. (Also spell out Johannesburg in Table 3 instead of JHB.) | JHB spelled in full |
| 35. Please created a Table of participant demographics (gender, age, role) as much as possible. | Table 4 has summarising the participant demographics has been added to the manuscript. |
| 36. Describe how focus group discussion participants were recruited. | The text has been edited to specify how the participants for the focus group discussion were selected:  “The data collectors facilitated nine focus group discussions with the CHWs recruited from the seven study sites. Every CHW who formed part of the CHW programme in these sites was invited to participate in the FGD. Where the CHWs exceeded the number suitable for a focus group discussion (7-10 participants), the researchers held two focus groups to accommodate the excess. There were no recorded refusals. Each FGD had approximately 10 participants, all women”. |
| 37. Minor: Line 158 - were should be was | “were” has been replaced with “was” |
| 38. Clarify the difference between the in-depth interviews and key informant interviews. | Key informant interviews have been deleted. Text has been integrated into in-depth interviews section |
| 39. The descriptors in Table 3 and then the two sections describing them are unclear; please give more insight into how these were differentiated or collapse the categories together. | We had difficulties in understanding what the reviewer meant by this comment. The first column of table 3 describes data collection methods (e.g. focus group discussion), while the other columns specify numbers of achieved per study site. It is not clear why one would want to collapse two different data collection methods into one row, or merge the numbers for the different sites. |
| 40. Clarify the consent processes for non-English speakers/readers, as you indicated that written consent was given prior to all research activities and that all instruments were in English. | To obtain participant consent, the data collectors explained the study purpose in English. Where participants struggled to understand or converse in the language, the data collectors communicated the information in either IsiZulu or Sotho accommodate the participants.  The following text in the paper has been strengthened to capture the above given explanation.  “The data collection instruments were in English, however where participants struggled to understand the questions or express themselves in English, the team provided clarity using local languages (Isizulu, Sesotho and Sepulana, an unofficial language spoke in some parts of Mpumalanga province).” |
| 41. Delete lines 188-190 - This introduction does not add to the section | Text has been deleted. |
| 42. Lines 203-206 seem out of place and are unclear. Please add further explanation, incorporate into other sections, or delete. | Text has been deleted. |
| 43. Line 227 - Define the timeframe; you indicate "several years," and we learn elsewhere that UHC CHW programs began in 2011 and the data were gathered from 2016-19. What time period are you referring to here? | Thank you for the comment.  South African community-based care programmes started prior to 2011 when the nation-wide CHW programme was introduced. The following text has been added to the paper.  “In Johannesburg, community orientated care was introduced by family medicine practitioners in one community health centre prior to 2011. The practice is called Chiawelo Community Practice and operate within the community health centre. It also functions as one of the local universities teaching and research platform.” |
| 44. Line 256 - Define RDP (only mention in paper) | RDP housing has been defined using a footnote  “These are houses that have been built by the government and are given to low-income families, as part of the Reconstruction and Development Programme (RDP) introduced in 1994”. |
| 45. Line 260 - Reword for judgement-neutral language, such as "residents faced challenges in maintaining medication protocols for chronic conditions." Minor: | We agree with the suggested text, changes to the paper has been made:    “The CHWs prioritized providing care to patients located in the informal settlements, as many of the residents faced challenges in maintaining medication protocols for chronic conditions” |
| 46. Line 333 - Change make to makes | Change made to the text |
| 47. Line 380: Define OTL (only mention in paper) | The term has been defined in the introduction part of the paper:  “the role of the outreach team leader (OTL) is to provide field supervision to the CHWs during household visits, ensure they (CHWs) have the resources that they require (e.g. stationary) to fulfil their duties, and help establish team relationship with community structures” |
| 48. Throughout the results section, it was challenging to follow the positionality of the respondents and data, since there are CHWs themselves, other health providers, other health system stakeholders, and community members included. Please clarify positions and differing views represented in your data based on role throughout sections. | We have provided more information on the positionality of the participants in the methods.  Furthermore, the abbreviated keys used in the findings (e.g. KI = key informant) is described in full at the end of the paper as per the requirement of the journal. |
| 49. **Discussion**   Overall, I felt like I was learning new things in the discussion that were not mentioned in the results. While the discussion should be a fresh analysis of your data, you should not be presenting new data in this section.  Line 442 - You mention medical doctors providing training for some CHWs. Make sure this is described in the results. Is this necessarily desirable/a programmatic goal? | Thank you for the feedback on the discussion. We carefully went through the discussion to ensure the only later presented under findings is discussed. We realised some of the information may not have been clearly presented in the findings  Yes – having doctors as part of a multi-disciplinary team who provide support and training to the CHW is integral to the COPC approach. This is illustrated in the text below that is from the findings. We have edited this text to so that it is clear that clinicians include medical doctors:  “In the urban sites, the CHWs received expert advice and mentorship from clinicians (e.g. medical doctors) and social services professionals in order to provide health promotion, prevention, screening services and referral for a wide range of health conditions. In Tshwane district, the CHWs met with members of a multi-disciplinary team weekly to debrief and receive case guidance. A key informant commented: “*Last week, I attended a meeting in the X clinic where the CHWs and Outreach Team Leader had a review meeting. The multi-disciplinary team consisting of a dietician, doctors, nurses and so forth attended the meeting. They reviewed the difficult cases and advised the CHWs accordingly”* (KI, interview, Tshwane). Similarly, the Sedibeng and Johannesburg teams had the support of medical doctors, nurses and clinical associates. The multi-disciplinary approach benefited the CHWs, as they had access to clinical knowledge shared during the meetings. In this meetings, the CHWs were able to present complex cases had to deal with in the field and receive expert advice” |
| 50. Line 444 - You use the loaded phrase "volatile communities," though this notion is not presented in the data. What data support this conclusion? How does the CHW program help or is it hampered by a specific set of community conditions? | We added more data demonstrating the volatility of the communities and how this impact the delivery of health services in the areas.  “Furthermore, in Ehlanzeni Team 6, a community representative who also served as a member of the clinic committee ensured the clinic was always represented during community meetings she addressed. This leader’s initiative helped ensure service delivery and health concerns of the residents were discussed in the same platform. However, community members were only interested in topics such as access to employment, water and decent housing. Many of the communities were politically volatile, with residents regularly protesting for decent housing and running water. In Sedibeng district, a health post was burned down by members of the community protesting against continuous electricity cuts in the area, while in another unrelated event district health officials were held hostage by CHWs protesting against poor work conditions (e.g. low stipend). These on-going service delivery protests often disrupted the engagements between the health team and communities on the days of the protests they can’t access the communities: “*We often have to stop work, because of service delivery protests*” (KI, Tshwane). |
| 51. Line 452-454 - It is great that you loop back to some of the literature. However, in the Discussion, you must directly link or compare to your results. What is our takeaway comparison to the Brazil literature? | Thank you for this feedback. We have critically reviewed through the discussion section and ensured key topics related to the research question are discussed in relation to literature covering various countries including Brazil.    Furthermore, we identified a systematic review of Brazil CHWs published in 2011, the review acknowledges the role of CHWs in extending access to basic health care (e.g. chronic care, maternal and child health). Poor skills mix, limited supervision, poor funding, remuneration of CHWs and political commitment limit the reach and effectiveness of the programmes.  We found the South African CHW programme is experiencing similar challenges. Using other related papers, we have reflected on the Brazil experience in the discussion and related this to the South African experience, see paragraph 2 of the discussion. |
| 52. Line 458 - How frequently did CHWs rarely interact with facility-based providers? I know it is a qualitative dataset, but please give us a sense of proportionality in the results. | Across the different sites, CHWs interaction with facility based staff members varied. CHWs in urban sites met with their supervisors and facility staff more frequently, while those in rural sites hardly met their supervisors and facility staff due to distance from their homes and community to the health facility.  The below text from the manuscript summarise the nature of interaction between CHWs and facility-based staff in the different sites.  “The CHWs in Team 2, led by a senior supervisor, received sufficient support from facility-based staff members, as the supervisor negotiated for their inclusion in facility activities and borrowed equipment (e.g., blood pressure machines) on their behalf. In order to build a good relationship with clinic staff, the supervisors in Johannesburg helped the nurses in the clinic: “*to ensure the patients referred by my people (CHWs) are attended timeously, I make time to assist the nurses in the clinic. I sometimes assist X in the triage room or attend women who are visiting the clinic for family planning services*” (CHW supervisor, interview, Team 3). However, support for CHWs planned activities was not forthcoming: “*I usually go to the facility manager’s office and inform her that I am having a campaign based on HIV, TB, vitamin A or de-worming. She will be happy but there will be no assistance, even if I can ask for assistance from the EPI or TB side. They will say, you are independent, you must know these things*” (CHW supervisor, interview, Team 5).  In the rural sites, when the CHW were at the clinic, they used a separate building, not the main clinic; this arrangement isolated the CHWs from the rest of the facility staff members. The team leaders also spent less time with the CHWs, as they only visited the facilities on Fridays. Between Monday and Thursday, from their homes they reported to the community. This was decided after it was found the CHWs spent more time walking between their homes, facility and community instead of providing care”. |
| 53. **Conclusion**   In both the Discussion and especially the Conclusion, you focus on lack of sustained funding as the downfall of CHW efforts. However, apart from the lapse of mobile devices, we do not get deep insight into this from your participants in the results. Make sure to focus the Discussion and Conclusion on the scope of your data. It is of course excellent for you to add your own analysis in these areas -- and funding cessation is clearly a key issue, but it was not the only issue raised in the data. | The authors have revisited the findings to add more data on the key topics that are discussed further in the discussion. Kindly see point 17 for the structure of the discussion and topics deliberated on in the discussion.  The conclusion has been edited to reflect on other topical issues such as the importance of supportive supervision and community participation:  “The implementation of community-orientated primary health care services is crucial in a resource constrained setting like South Africa. However, the implementation of these programmes often get derailed by socio-economic issues dominant in the communities. Poor community participation in the design and implementation of the programmes, limited health system support for outreach team and unsustained funds make it difficult for the teams to be effective in the delivery of care. In order to strengthen these programmes, CHWs need to be integrated into healthcare systems so to access supportive supervision, have access to resources, and government make available funding to implementing teams”. |
| **Reviewer 6** | |
| **Comments** | **Authors responses** |
| 54. data collection: 1. The authors say that data collection tools we in English: but the (data collection) team provided additional local language explanation/interpretation to the interview questions for participants who did not understand English. I would like the authors to comment on the views of possible varied explanations/interpretations of the interview questions (hence varied responses) due to lack of standardized translated interview guides. Related to this: I feel the authors need to acknowledge, include and reflect on lack of standardized translation of interview guided as part of the study limitations documented in page 20 | Thank you for this valuable feedback on the data collection tools. It is likely that the data collectors might have translated the interview questions slightly differently to participants due to the tools being in English. We have captured this as one of the limitations of the study.  “Third, the data collection tools were in English, this might have resulted in different explanation and interpretation of the interview questions during data collection”  We minimised the impact of lack of standardised translated interview guides by having the data collectors participate in role-plays where they would act as an interviewers and participants prior to data collection. The activity helped familiarise the collectors within the interview questions, develop ways to translate the interview questions to local languages in a consistent manner. |
| 55. The authors should include a brief section on recommendations/policy and practice implication of the study findings just before the conclusions | Several of the paragraphs in the discussion focus on the implications for policy, including issues around the funding of CHW programmes, the need to make supervision available and importance of community participation in the programmes |
| 56. The authors use both direct and indirect reporting styles. E.g. In the abstract “…… In this article, we explore to what extent a nation-wide CHW programme in South Africa is attuned to community needs, integrated into the healthcare….” ( direct reporting) and “…..e study was conducted in seven primary healthcare facilities located in semi-urban and rural …” (indirect reporting). There is need to stick to one style all through. | We prefer to use direct reporting. We have checked this and ensured that this is consistent throughout the manuscript. |
| 57. The authors refer to data in singular ( “ data was” ) in various places in the methods section. Data should be referred to in plural (“Data were”) | Corrected throughout the manuscript |
| 58. Page 12: “a mobile app” should be written in full as “ a mobile phone application” | Mobile app written in full |
| 59. Page 18 line 449 : “A review in low and middle income countries has found …..” . Should be: “A review of literature from low and middle income countries has found …” | Sentence corrected |

Kind regards,

Hlologelo Malatji
